# Impact of Dysmenorrhea Severity on Mental Wellbeing and Quality of Life Among Saudi Women: A Cross-Sectional Study

**DOI:** 10.3390/medicina62010004

**Published:** 2025-12-20

**Authors:** Ghadeer A. Alneel, Mohammad A. Jareebi, Dhiyaa A. H. Otayf, Saja A. Almraysi, Raimaa A. Alhassan, Areej H. Zughaibi, Seba Y. Muzaiiadi, Altaf A. Abdulhaq, Maha H. Alzubair, Huda A. Alramadhan, Khalid M. Akkour, Adhari A. Alselmi, Farjah H. Algahtani, Hani A. Alghamdi, Ghazi I. Al Jowf

**Affiliations:** 1 Department of Obstetrics & Gynaecology, Women’s Health Hospital, Riyadh 14611, Saudi Arabia; ghadeer-ahmad-n@hotmail.com (G.A.A.); mahahmeddr@gmail.com (M.H.A.); 2 Family and Community Medicine Department, Faculty of Medicine, Jazan University, Jazan 45142, Saudi Arabia; 3 Faculty of Medicine, Jazan University, Jazan 45142, Saudi Arabia; dhiyaaot@gmail.com (D.A.H.O.); sajaalasiri1@gmail.com (S.A.A.); dr.raimaa@gmail.com (R.A.A.); seba.muzaiiadi@gmail.com (S.Y.M.); 4 Department of Obstetrics and Gynecology, Saudi Ministry of Health, Riyadh 11176, Saudi Arabia; ahzughaibi@moh.gov.sa; 5 Faculty of Pharmacy, Jazan University, Jazan 45142, Saudi Arabia; altaaaf2018@gmail.com; 6 Dawadmi General Hospital, Dawadmi 17463, Saudi Arabia; halramadhan8520@gmail.com; 7 Obstetrics and Gynecology Department, Faculty of Medicine, King Saud University, Riyadh 11451, Saudi Arabia; khalidakkour@gmail.com; 8 Clinical Sciences Department, MBBS Program Fakeeh College for Medical Sciences, Jeddah 21461, Saudi Arabia; adhari.alselmi@hotmail.com; 9 Department of Medicine, Oncology Center, Chair of Epidemiology and Public Health Research, Faculty of Medicine, King Saud University/King Saud Medical City, Riyadh 12373, Saudi Arabia; falgahtani@ksu.edu.sa; 10 Department of Family and Community Medicine, College of Medicine, King Saud University, Riyadh 12373, Saudi Arabia; halhajalah@ksu.edu.sa; 11 Department of Public Health, College of Applied Medical Sciences, University Medical Clinics Complex, King Faisal University, Al Hofuf, Al Ahsa 37912, Saudi Arabia; galjowf@kfu.edu.sa

**Keywords:** dysmenorrhea, pain severity, mental health, quality of life, women health, Saudi Arabia

## Abstract

*Background and Objectives:* Dysmenorrhea affects 50–90% of women worldwide and significantly impacts quality of life. This study aimed to determine the nationwide prevalence of dysmenorrhea among Saudi women and evaluate the independent associations between pain severity, associated symptoms, and mental health and quality of life outcomes. *Materials and Methods:* A cross-sectional study was conducted between May and August 2024 among women aged 18–55 years in Saudi Arabia. Data were collected through an online survey assessing sociodemographic characteristics, menstrual patterns, dysmenorrhea severity (Visual Analogue Scale, VAS 1–10), associated symptoms, mental health (DASS-21), and quality of life (MQLI). Univariate comparisons and multiple linear regression analyses were performed to identify independent predictors of depression, anxiety, stress, and quality of life (QoL). *Results:* Of 950 women (mean age 28 ± 9.5 years, BMI 24 ± 5.8 kg/m^2^), 87% reported dysmenorrhea, with 50% experiencing pain every cycle and 55% reporting severe pain (VAS 7–10). Women with severe pain exhibited depression scores 47% higher than those with mild pain (21.8 vs. 14.8, *p* < 0.001), with similar patterns for anxiety and stress. In multivariate analyses, severe pain (VAS 8–10) was associated with 7–11-point increases in DASS scores (all *p* < 0.001). Constipation emerged as the strongest symptom-related predictor of depression (β = 4.94, *p* < 0.001), anxiety (β = 4.79, *p* < 0.001), stress (β = 3.96, *p* < 0.001), and reduced quality of life (β = −0.45, *p* = 0.015). Risk factors included having children, higher BMI, and longer menstrual cycles, while higher income, later menarche, and greater education were protective. *Conclusions:* Pain severity, not dysmenorrhea presence alone, drives mental health burden. Constipation represents a novel therapeutic target. Integrated care addressing pain, gastrointestinal symptoms, and mental health is essential.

## 1. Introduction

Dysmenorrhea, characterized by painful menstruation, is a highly prevalent gynecological condition affecting 50% to 90% of reproductive-aged women globally [1]. This condition constitutes a major public health concern due to its high prevalence and marked impact on quality of life, particularly among young women. In Saudi Arabia, regional studies report alarming prevalence rates, with 81% of medical students in Riyadh experiencing primary dysmenorrhea and 74.4% among school students in Northern Saudi Arabia [2]. A more recent nationwide study found that 92% of Saudi women reported primary dysmenorrhea, while 7% had secondary dysmenorrhea [3].

Dysmenorrhea is classified into primary and secondary types, each with distinct etiologies and clinical presentations. Primary dysmenorrhea refers to menstrual pain without underlying pelvic pathology and usually emerges around the onset of menarche [4]. It is cramping lower abdominal pain lasting 8–72 h. Conversely, secondary dysmenorrhea is associated with underlying pelvic disorders including endometriosis, fibroids, or adenomyosis, and often manifest later in reproductive life with increasing pain severity and non-cyclical symptoms [5]. Studies indicate that young age (<25 years), early menarche (<12 years), nulliparity, irregular or heavy bleeding, and psychological stress are key risk factors for dysmenorrhea among Saudi women. Additionally, obesity (BMI ≥ 30) and sedentary lifestyle further elevate the risk, particularly in urban populations [6].

Beyond physical pain, dysmenorrhea significantly impairs emotional and social functioning. Studies indicate that 20.1% of affected women experience school absenteeism, while 40.9% report reduced academic performance [7]. The condition correlates with poorer sleep quality, higher psychological distress, and diminished social interactions, often surpassing the burden of chronic illnesses such as cystic fibrosis [3,8]. Emerging evidence suggests that dysmenorrhea severity, rather than mere presence, may be the primary driver of these adverse outcomes. However, the relationship between pain intensity and mental health remains inadequately characterized, particularly regarding dose-response patterns and the role of associated symptoms such as gastrointestinal disturbances.

Despite its widespread occurrence and severe consequences, dysmenorrhea is often underreported and undertreated in Saudi Arabia, leading to significant gaps in research and clinical management. Most studies in Saudi Arabia are localized, with limited generalizability to the broader population. Given Saudi Arabia’s large female population—approximately 8 million across 13 regions [9]—there is a clear need for comprehensive epidemiological data on dysmenorrhea. Furthermore, while previous research has documented dysmenorrhea prevalence and basic associations with quality of life [3], few studies have systematically examined the independent contribution of pain severity versus associated symptoms to mental health outcomes or identified specific risk and protective factors that could inform targeted interventions.

Therefore, this study aimed to determine the nationwide prevalence of dysmenorrhea among Saudi women, assess pain severity distribution and frequency, identify associated determinants, and evaluate the independent associations between dysmenorrhea characteristics—particularly pain severity and associated symptoms—and mental health outcomes (depression, anxiety, stress) and quality of life. The findings are expected to guide evidence-based public health strategies, inform clinical practice regarding screening and integrated management approaches, and identify high-risk populations requiring targeted support.

## 2. Materials and Methods

### 2.1. Study Design and Setting

A cross-sectional study was conducted in the Kingdom of Saudi Arabia between May and August 2024. The inclusion criteria comprised all adult women aged 18 to 55 years who were residents of Saudi Arabia and provided informed consent. Women younger than 18 years or older than 55 years, as well as those unwilling to provide consent, were excluded from the study.

### 2.2. Sampling and Sample Size Calculation

Participants were recruited using a convenience sampling technique through online platforms. According to the most recent national census, 8,768,559 females between the ages of 18 and 55 years comprised the target population [9]. Using a 95% confidence level, a 4.5% margin of error, and accounting for a 20% non-response rate, the minimum required sample size was calculated as 950 participants, according to the following formula:

$$n_{o}=\frac{Z^{2}\cdot p\cdot (1-p)}{e^{2}}$$

where *Z* denotes the standard score corresponding to the selected confidence level (1.96 for a 95% Cl), *p* indicates the anticipated prevalence or proportion (commonly 0.5 for a conservative estimate), and *e* represents the allowable margin of error [10].

### 2.3. Data Collection Tool

An online self-administered questionnaire was developed and distributed through social media platforms, including WhatsApp and Facebook. The questionnaire comprised four main sections: (1) sociodemographic characteristics, (2) menstrual cycle patterns and dysmenorrhea characteristics, (3) mental health assessment, and (4) quality of life evaluation.

#### 2.3.1. Section 1: Sociodemographic and Health Characteristics

The sociodemographic section collected information on age, body mass index (BMI, calculated from self-reported height and weight), employment status, marital status, number of children, educational attainment, monthly income level, and residence (urban or rural). Additional health-related information included smoking status and type (cigarettes, shisha/hookah/waterpipe/vape), physical activity patterns (yes/no), history of hemoglobinopathies (sickle cell anemia, thalassemia), and history of gynecological surgical procedures.

#### 2.3.2. Section 2: Menstrual Cycle and Dysmenorrhea Characteristics

The second section assessed menstrual cycle characteristics, including age at menarche, menstrual cycle length (in days), bleeding duration (in days), menstrual blood flow amount (light, moderate, or heavy based on sanitary pad saturation), and period regularity. Dysmenorrhea was assessed through a binary question asking participants whether they experienced menstrual pain (yes/no). For women reporting dysmenorrhea, additional questions captured:

Pain severity: Measured using a Visual Analogue Scale (VAS) ranging from 1 (minimal pain) to 10 (worst imaginable pain).

Pain frequency: Categorized as occurring once every six months, once every three months, or in every menstrual cycle.

Pain timing: Onset relative to menstruation (2–3 days before, first day, or second-third day of menstruation).

Pain duration: Categorized as one day, two to three days, or more than three days.

Pain management: Methods used to include no medication, over-the-counter medication, or emergency department visits.

Healthcare utilization: History of emergency department visits for dysmenorrhea and their frequency.

Associated symptoms: Presence of abdominal bloating, mood swings, diarrhea, and constipation during menstruation.

#### 2.3.3. Section 3: Mental Health Assessment

Mental health was evaluated using the Arabic version of the Depression, Anxiety, and Stress Scale-21 (DASS-21), a validated self-report instrument [11]. DASS-21 consists of three subscales, each containing seven items that assess depression, anxiety, and stress. Participants rated each item on a 4-point Likert scale (0 = did not apply to me at all, 3 = applied to me very much or most of the time). Scores for each subscale were calculated by summing the responses for the seven items and then multiplying the total by two, yielding a possible range of 0 to 42 for each domain. Higher scores indicated greater symptom severity. Cut-off values for severe symptoms were defined as scores above 20 for depression, above 14 for anxiety, and above 25 for stress [12]. Additionally, severity categories were classified as follows: for depression (Normal: 0–9, Mild: 10–13, Moderate: 14–20, Severe: 21–27, Very Severe: ≥28); for anxiety (Normal: 0–7, Mild: 8–9, Moderate: 10–14, Severe: 15–19, Very Severe: ≥20); and for stress (Normal: 0–14, Mild: 15–18, Moderate: 19–25, Severe: 26–33, Very Severe: ≥34).

#### 2.3.4. Section 4: Quality of Life Assessment

Quality of life was assessed using the Multicultural Quality of Life Index (MQLI), a validated 10-item self-report instrument that evaluates physical, psychological, social, and environmental well-being [13]. Each item was rated on a scale from 1 (very poor) to 10 (excellent), with higher scores reflecting better quality of life. The overall QoL score was calculated as the mean of all 10 items.

### 2.4. Statistical Analysis

Following data collection, the raw dataset was transferred to Microsoft Excel for preliminary data cleaning, error detection, and validation. Subsequent statistical analyses were performed using R software (version 4.2.3; R Foundation for Statistical Computing, Vienna, Austria). Continuous variables were presented as means ± standard deviations (SD), while categorical variables were presented as frequencies and percentages. Pain severity was categorized into three groups: mild (VAS 1–3), moderate (VAS 4–6), and severe (VAS 7–10). Univariate associations between dysmenorrhea characteristics and mental health outcomes were assessed using independent t-tests for binary comparisons and one-way ANOVA for pain severity categories. Spearman correlation coefficients were calculated to examine relationships between pain severity and mental health scores. Multiple linear regression analyses were performed to identify independent predictors of depression, anxiety, stress, and quality of life. Models included dysmenorrhea-related variables (pain severity with VAS 1 as reference, age at menarche, cycle length, period regularity, associated symptoms, emergency visits, and gynecological surgery history) and sociodemographic covariates (age, marital status, income, education, employment, number of children, residence, smoking, and BMI). Statistical significance was set at *p* < 0.05 (two-tailed).

### 2.5. Ethical Approval

This study received ethical clearance from the Standing Committee for Scientific Research at Jazan University (Approval No. REC-45/11/1115, dated 28 May 2024). Prior to participation, all individuals were informed about the study objectives, potential outcomes, and their rights, including voluntary participation and confidentiality protections. Electronic informed consent was obtained from all participants before accessing the online questionnaire. To safeguard participant privacy, no personal identifiable information was collected, and all data was anonymized and stored securely with access restricted exclusively to authorized members of the research team. The study adhered to the ethical principles outlined in the Declaration of Helsinki and followed the Strengthening the Reporting of Observational Studies in Epidemiology (STROBE) guidelines to ensure methodological rigor and transparent reporting of this cross-sectional investigation.

### 2.6. Use of Generative Artificial Intelligence

Generative AI was used solely for language editing. Study design, data, analysis, and interpretation were entirely conducted by the author.

## 3. Results

### 3.1. Participant Characteristics

This study included 950 female participants with a mean age of 28 ± 9.5 years and a mean body mass index (BMI) of 24 ± 5.8 kg/m^2^. More than half of the participants held a bachelor’s degree (53%), while 36% had a high school degree or lower, and 11% had completed postgraduate studies. Regarding employment status, 34% were employed, 48% were students, and 18% were unemployed. Over half were unmarried or engaged (55%), while 41% were married and 4% were divorced or widowed. Sixty percent reported having no children, whereas 10% had one child, 12% had two, and 18% had three or more. In terms of income, 59% earned less than 5000 SR monthly, while 19% reported monthly earnings between 5000 and 9999 SR, and 11% earned between 10,000 and 14,999 SR. The majority of participants resided in urban areas (79%) (Table 1).

### 3.2. Health Status and Lifestyle Factors

Among the participants, 5% reported smoking, with cigarette use accounting for 1% and shisha/hookah/waterpipe use accounting for 5%. Approximately 35% reported being physically active. With respect to medical history, 11% had sickle cell anemia and 7% had thalassemia. Additionally, 17% had undergone gynecological surgery (Table 2).

### 3.3. Menstrual Cycle Characteristics

**Figure 1:**
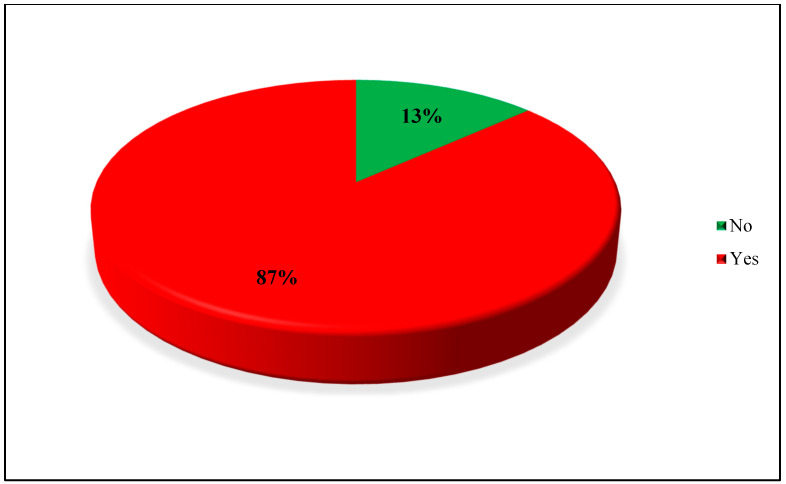
Prevalence of dysmenorrhea among participants.

The mean age at menarche was 12 ± 2 years, with a mean cycle length of 27 ± 7.4 days and a mean menstrual bleeding duration of 5.2 ± 2.2 days. Regarding menstrual blood flow, 10% of participants reported heavy bleeding (more than 1 fully soaked sanitary pad every 2 h), 62% reported moderate bleeding (more than 1 soaked sanitary pad within 3 h), and 28% reported light bleeding (less than 1 soaked pad in 3 h). The majority of participants (86%) reported regular menstrual periods (Table 3). Dysmenorrhea was reported by 830 participants (87%) (Figure 1).

### 3.4. Dysmenorrhea Characteristics and Associated Symptoms

Among women reporting dysmenorrhea (n = 830), pain intensity was assessed using a visual analogue scale (VAS) ranging from 1 to 10. The distribution of pain severity revealed that 14% experienced mild pain (VAS 1–3), 38% experienced moderate pain (VAS 4–6), and 48% experienced severe pain (VAS 7–10). The most reported pain score was 7 (21% of participants), followed by scores of 8 (16%) and 5 (16%). Notably, 26% of participants reported severe pain at the upper end of the scale (VAS 7–10 combined), with 5% rating their pain as 10/10. Regarding pain frequency, half of the participants (50%) experienced pain during every menstrual cycle, while 28% reported pain in some cycles (once every three months), and 22% experienced pain infrequently (once every six months). Pain management strategies varied: 50% used over-the-counter medication, 44% did not use any medication, and 6% required emergency department visits. Overall, 15% of participants reported needed emergency care for dysmenorrhea at some point, with 11% visiting once, 6% visiting twice, and 7% visiting three times or more. Pain onset typically occurred on the first day of the menstrual cycle (42%), followed by 2–3 days before menstruation began (39%), and during the second or third day of menstruation (11%). Pain duration was most commonly 2–3 days (49%), followed by one day (37%), and more than 3 days (8%). The most frequently reported associated symptoms included mood swings (79%), abdominal bloating (60%), diarrhea (31%), and constipation (27%) (Table 4).

### 3.5. Mental Health and Quality of Life Scores

Mental health was assessed using Depression, Anxiety, and Stress Scale (DASS-21). The mean depression score was 19 ± 12, mean anxiety score was 16 ± 11, and mean stress score was 22 ± 12. Quality of life, measured using the Multicultural Quality of Life Index (MQLI), had a mean score of 5.7 ± 2.4 out of 10. When categorized by severity, 26% of participants reported normal depression levels, while 31% experienced very severe depression. For anxiety, 33% fell within the normal range, while 20% experienced very severe anxiety. Stress showed the highest burden, with only 18% reporting normal levels and 43% experiencing very severe stress (Table 5).

### 3.6. Univariate Associations Between Dysmenorrhea and Mental Health Outcomes

To examine the relationship between dysmenorrhea characteristics and mental health outcomes before adjusting for confounders, we conducted univariate comparisons using independent t-tests and one-way ANOVA. Women with dysmenorrhea had significantly higher depression scores (19.6 ± 12.3 vs. 15.2 ± 10.5, *p* = 0.001), anxiety scores (16.5 ± 11.2 vs. 13.8 ± 9.8, *p* = 0.018), and stress scores (22.5 ± 12.1 vs. 19.1 ± 11.2, *p* = 0.006) compared to women without dysmenorrhea. Quality of life scores were lower among women with dysmenorrhea, though this difference did not reach statistical significance (5.6 ± 2.4 vs. 6.1 ± 2.2, *p* = 0.062). A strong dose-response relationship emerged when examining pain severity categories. Women with severe pain (VAS 7–10) exhibited depression scores 47% higher than those with mild pain (VAS 1–3) (21.8 ± 12.6 vs. 14.8 ± 10.8, *p* < 0.001), anxiety scores 39% higher (18.4 ± 11.5 vs. 13.2 ± 9.5, *p* < 0.001), and stress scores 29% higher (24.2 ± 12.3 vs. 18.7 ± 10.9, *p* < 0.001). Quality of life declined progressively with increasing pain severity (mild: 6.2 ± 2.3; moderate: 5.9 ± 2.4; severe: 5.3 ± 2.4, *p* < 0.001). Pain frequency also showed significant associations. Women experiencing pain every menstrual cycle had higher depression (21.1 ± 12.4 vs. 17.2 ± 11.8, *p* < 0.001), anxiety (17.5 ± 11.6 vs. 14.9 ± 10.5, *p* < 0.001), and stress scores (23.8 ± 12.3 vs. 20.5 ± 11.6, *p* < 0.001) compared to those with occasional pain. Similarly, women who required emergency department visits for dysmenorrhea demonstrated significantly higher depression (22.7 ± 13.1 vs. 18.5 ± 11.9, *p* < 0.001), anxiety (18.9 ± 11.8 vs. 15.6 ± 10.9, *p* = 0.003), and stress scores (24.5 ± 12.5 vs. 21.7 ± 12.0, *p* = 0.018), along with lower quality of life (5.2 ± 2.3 vs. 5.8 ± 2.4, *p* = 0.010) (Table 6).

#### Correlation Between Pain Severity and Mental Health Outcomes

To further quantify the relationship between pain severity and mental health, we examined Spearman correlation coefficients. Pain severity showed moderate positive correlations with depression (r = 0.42, *p* < 0.001), anxiety (r = 0.45, *p* < 0.001), and stress (r = 0.40, *p* < 0.001), indicating that higher pain intensity was consistently associated with worse mental health outcomes. Pain severity also showed a significant negative correlation with quality of life (r = −0.28, *p* < 0.001), demonstrating that more severe pain was associated with poorer quality of life. These correlations remained significant even when examining only women with dysmenorrhea (n = 830), confirming that the relationship between pain severity and mental health outcomes exists independently of dysmenorrhea diagnosis.

### 3.7. Multivariate Analysis: Independent Predictors of Mental Health and Quality of Life

To identify independent dysmenorrhea-related predictors of mental health and quality of life while controlling for sociodemographic, lifestyle, and health factors, we performed multiple linear regression analyses. Table 7 presents the adjusted associations for dysmenorrhea-related variables.

#### 3.7.1. Pain Severity and Dose-Response Relationship

Pain severity demonstrated a consistent dose-response relationship across all mental health domains (Table 7). Compared to mild pain (VAS 1–3), moderate pain (VAS 4–6) was associated with modest increases in depression (β = 3.27, 95% CI: 0.38–6.16, *p* = 0.027) and anxiety (β = 2.64, 95% CI: −0.09–5.37, *p* = 0.058), though not consistently significant across all outcomes. However, severe pain (VAS 7–10) showed robust associations with substantially elevated mental health symptoms. Specifically, the highest pain scores (VAS 8–10) were associated with 7–11-point increases in depression scores: VAS 8 (β = 7.60, 95% CI: 3.40–11.80, *p* < 0.001), VAS 9 (β = 10.95, 95% CI: 6.19–15.72, *p* < 0.001), and VAS 10 (β = 8.19, 95% CI: 3.19–13.19, *p* = 0.001). Similar patterns emerged for anxiety, with VAS 8 (β = 6.64, 95% CI: 2.92–10.36, *p* < 0.001), VAS 9 (β = 9.95, 95% CI: 5.73–14.17, *p* < 0.001), and VAS 10 (β = 9.16, 95% CI: 4.73–13.59, *p* < 0.001). For stress, severe pain scores showed comparable associations: VAS 8 (β = 6.60, 95% CI: 2.55–10.66, *p* = 0.001), VAS 9 (β = 10.34, 95% CI: 5.74–14.94, *p* < 0.001), and VAS 10 (β = 8.99, 95% CI: 4.17–13.82, *p* < 0.001). For quality of life, the impact of pain was most pronounced at moderate pain levels, with VAS scores 3–6 associated with approximately 0.9–1.1-point decreases in QoL scores (VAS 3: β = −1.14, *p* = 0.024; VAS 5: β = −0.89, *p* = 0.046; VAS 6: β = −0.97, *p* = 0.032).

#### 3.7.2. Dysmenorrhea-Associated Symptoms

Among dysmenorrhea-associated symptoms, constipation emerged as the strongest independent predictor across all outcomes, with effect sizes comparable to severe pain. Constipation was associated with substantially elevated depression (β = 4.94, 95% CI: 3.27–6.62, *p* < 0.001), anxiety (β = 4.79, 95% CI: 3.31–6.27, *p* < 0.001), and stress scores (β = 3.96, 95% CI: 2.35–5.58, *p* < 0.001), as well as reduced quality of life (β = −0.45, 95% CI: −0.81 to −0.09, *p* = 0.015). Abdominal bloating showed marginal associations with anxiety (β = 1.28, 95% CI: −0.03–2.59, *p* = 0.056) and significantly reduced quality of life (β = −0.32, 95% CI: −0.64 to −0.00, *p* = 0.050). Mood swings and diarrhea were not independently associated with mental health outcomes after adjusting for other variables (all *p* > 0.05).

#### 3.7.3. Healthcare Utilization and Gynecological History

Emergency department visits for dysmenorrhea were not significantly associated with mental health outcomes after adjusting for pain severity and other factors (all *p* > 0.05). However, past gynecological surgery was associated with increased anxiety (β = 1.97, 95% CI: 0.28–3.65, *p* = 0.022) and reduced quality of life (β = −0.52, 95% CI: −0.93 to −0.11, *p* = 0.013).

#### 3.7.4. Menstrual Cycle Characteristics and Mental Health

Among menstrual characteristics, later age at menarche emerged as a protective factor against depression (β = −0.48, 95% CI: −0.83 to −0.13, *p* = 0.007), anxiety (β = −0.32, 95% CI: −0.63 to −0.02, *p* = 0.040), and stress (β = −0.51, 95% CI: −0.85 to −0.17, *p* = 0.003). Each additional year of delay in menarche onset was associated with approximately 0.3–0.5-point reductions in mental health symptom scores. Longer menstrual cycle length was associated with higher depression (β = 0.17, 95% CI: 0.07–0.27, *p* = 0.001), anxiety (β = 0.11, 95% CI: 0.02–0.20, *p* = 0.012), and stress scores (β = 0.20, 95% CI: 0.10–0.29, *p* < 0.001), though not with quality of life. Period regularity was not significantly associated with any outcome after adjustment (all *p* > 0.05) (Table 7).

## 4. Discussion

### 4.1. Principal Findings

Dysmenorrhea is one of the most prevalent gynecological conditions globally, affecting 50–90% of reproductive-aged women. The present study explored the nationwide prevalence, severity, and impact of dysmenorrhea among Saudi women, with specific focus on mental health and quality of life outcomes. Our findings revealed that 87% of participants experienced dysmenorrhea, with half reporting pain during every menstrual cycle and 55% experiencing severe pain (VAS 7–10). A critical finding emerged regarding the relationship between dysmenorrhea and mental health. While women with dysmenorrhea showed higher depression (19.6 vs. 15.2, *p* = 0.001), anxiety (16.5 vs. 13.8, *p* = 0.018), and stress scores (22.5 vs. 19.1, *p* = 0.006) in univariate analyses, multivariate models revealed that pain severity, rather than dysmenorrhea presence alone, was the primary driver of psychological distress. Women with severe pain (VAS 7–10) exhibited depression scores 47% higher than those with mild pain, with similar patterns for anxiety and stress. The highest pain scores (VAS 8–10) were associated with 7–11 point increases in DASS scores after adjusting for confounders, representing clinically significant elevations in mental health symptoms. These findings suggest that the burden of dysmenorrhea—particularly pain intensity and associated symptoms—rather than menstrual pain per se, drives psychological distress. The inability to distinguish between primary and secondary dysmenorrhea is an essential consideration that may have significantly affected the study results. Notably, 17% of participants had a history of gynecological surgery, depicted that a substantial proportion may have experienced secondary dysmenorrhea, which has distinct etiologies, underlying causes, and clinical management compared to primary dysmenorrhea. This overlap complicates the interpretation of observed risk factors and symptom severity, as some associations may reflect characteristics of secondary dysmenorrhea rather than primary. As a result, the overall burden contributed to primary dysmenorrhea could be overestimated. Interpretation of this distinction in future studies is important to accurately assess prevalence, identify specific risk factors, and guide targeted interventions for each type.

### 4.2. Prevalence and Severity of Dysmenorrhea

The observed prevalence of 87% is consistent with recent Saudi studies reporting 92.3% in Riyadh [2] and 83.7% among university students [14] and aligns with global estimates of 71–73% [15,16,17]. This difference may partly depict the sampling bias and the absence of differentiation between primary and secondary dysmenorrhea. These methodological factors likely accounted for an increased prevalence, in addition to demographic or cultural factors. In this study, half of participants experienced pain every cycle and 55% reported severe pain (VAS 7–10), comparable to international findings showing 43% with pain every cycle [18] and 2–32% with severe pain across populations [19,20,21]. The variability in pain severity depends on multiple interacting factors including age, BMI, menstrual flow, diet, physical activity, psychological distress, and prevalence of secondary conditions [22,23,24]. In Saudi Arabia, high rates of sedentary behavior, obesity (45.1% overweight, 23.1% obese) [25], reliance on herbal remedies (69.1%), and limited medical care-seeking (14.6%) may exacerbate symptoms and limit effective management [26,27,28].

### 4.3. Pain Severity and Mental Health: A Dose-Response Relationship

The most important finding of this study is the robust dose-response relationship between pain severity and mental health outcomes. Spearman correlations demonstrated moderate positive associations between pain severity and depression (r = 0.42), anxiety (r = 0.45), and stress (r = 0.40), all significant at *p* < 0.001. Previous studies have reported similar correlations between dysmenorrhea severity and depression (r = 0.216), anxiety (r = 0.207), and global psychological distress (r = 0.311) [29], with primary dysmenorrhea increasing risk of depressive disorder (RR = 1.72, *p* < 0.001) [30]. The mechanism likely involves recurrent cyclical pain triggering anticipatory anxiety, sleep disturbance, and circadian disruption, thereby amplifying pain sensitivity, mood dysregulation, and impaired daily functioning [31]. Literature indicates that depression, anxiety, and stress levels are elevated during menstruation compared with non-menstrual days, with symptoms frequently emerging in the premenstrual phase [32]. However, when adjusting for multiple confounders including workplace and gynecological variables, some studies found that dysmenorrhea-mental health associations became non-significant [33], underscoring the complex, multifactorial nature of this relationship.

### 4.4. Constipation: A Novel and Robust Predictor

Among dysmenorrhea-associated symptoms, constipation emerged as the strongest independent predictor across all mental health outcomes, with effect sizes comparable to severe pain itself. This finding represents an important contribution warranting clinical attention. Previous studies have established that constipation correlates significantly with depression (*p* = 0.001) [34] and confers 48% higher risk of developing depression (aHR 1.48; 95% CI, 1.41–1.56) [35]. The relationship between gastrointestinal function and mental health is bidirectional and mediated through the gut-brain axis. Hormonal fluctuations across the menstrual cycle, particularly progesterone drops around menstruation, slow gastrointestinal motility and cause constipation [36]. However, alterations in intestinal microbiota during constipation influence serotonin regulation—approximately 95% of serotonin is produced in the gastrointestinal tract—contributing to mood disturbances [37]. Current dysmenorrhea management focuses predominantly on pain relief, yet our findings suggest that addressing constipation through dietary fiber, hydration, probiotics, and physical activity may represent a novel therapeutic target for improving both physical symptoms and mental health outcomes.

### 4.5. Reproductive and Sociodemographic Risk Factors

Women with children, particularly one or two children, demonstrated significantly higher depression, anxiety, and stress scores compared to nulliparous women. This aligns with previous research showing 1.13-fold increased risk of postpartum depression among women with dysmenorrhea (OR = 1.13; 95% CI: 1.06–1.21) [37] and higher dysmenorrhea prevalence in women with postpartum depression (64.2% vs. 47.9%, *p* = 0.004) [38]. The relationship is likely bidirectional: depression and stress dysregulate the hypothalamic-pituitary-adrenal and hypothalamic-pituitary-ovarian axes, disrupting hormonal balance and elevating prostaglandin activity [39,40], while women with dysmenorrhea who become mothers face compounded stress from managing chronic pain alongside childcare responsibilities.

Higher BMI was positively associated with depression and anxiety, consistent with evidence that obesity, elevated BMI, and irregular menstrual cycles correlate with increased stress and depression (r = 0.482, *p* < 0.001) [41]. Obesity represents a chronic inflammatory condition with increased circulating inflammatory mediators implicated in mental disorders and delayed endometrial repair [42,43]. Longer menstrual cycle length was associated with higher depression, anxiety, and stress scores, potentially reflecting underlying hormonal irregularities or polycystic ovary syndrome.

### 4.6. Protective Factors and Quality of Life Impact

Several protective factors emerged from the multivariate analyses. Higher income (5000–9999 SR; β = 0.56, *p* = 0.025) and postgraduate education (β = 1.40, *p* < 0.001) were positively associated with quality of life, while higher education correlated with lower anxiety scores (bachelor’s: β = −2.06, *p* = 0.007; postgraduate: β = −4.60, *p* < 0.001). Previous literature demonstrates that higher education and household income confer significantly lower odds of depressive symptoms [44], likely through improved healthcare access, health literacy, and self-management strategies. Later age at menarche emerged as a protective factor against depression (β = −0.48, *p* = 0.007), anxiety (β = −0.32, *p* = 0.040), and stress (β = −0.51, *p* = 0.003). Although evidence regarding menarche timing and mental health is mixed—some studies report increased depression risk with later menarche [45] while others show higher risk with early menarche [46,47]—our finding may reflect that early menarche exposes adolescents to dysmenorrhea during a developmentally sensitive period when coping mechanisms are immature.

In contrast to mental health outcomes where severe pain showed the strongest associations, quality of life was most significantly impaired at moderate pain levels (VAS 3–6), potentially reflecting measurement sensitivity differences between the 10-point MQLI and 42-point DASS-21 scales. Constipation was associated with reduced quality of life (β = −0.45, *p* = 0.015), reinforcing its effects across both mental health and functional well-being. Additionally, abdominal bloating (β = −0.32, *p* = 0.050), gynecological surgery history (β = −0.52, *p* = 0.013), and higher BMI (β = −0.04, *p* = 0.008) were linked to poorer quality of life, suggesting that dysmenorrhea’s burden operates through multiple pathways requiring comprehensive management approaches.

Dysmenorrhea substantially impaired quality of life, with 15% requiring emergency care, reflecting inadequate outpatient management. Previous studies demonstrate significant impairments across multiple domains [29], with dysmenorrhea associated with 67.5% reduced productivity among healthcare workers [48] and disrupting students’ routine work (37.8%), sleep (29.2%), and social functioning (33.8%) [48]. Students with dysmenorrhea exhibit significantly lower quality of life scores (−1.82; 95% CI: −2.63 to −1.02; *p* < 0.001) [49], underscoring the need for routine menstrual health and mental health screening [50].

### 4.7. Clinical and Public Health Implications

Healthcare providers should shift focus from binary assessment to evaluating dysmenorrhea severity using validated pain scales, with women reporting pain scores ≥ 7 prioritized for aggressive pain management and mental health screening using instruments such as PHQ-9 or GAD-7. Integrated management addressing both physical symptoms and mental health is essential, recognizing that severe dysmenorrhea warrants psychological support alongside pharmacological treatment. Importantly, gastrointestinal symptom management should be integrated into dysmenorrhea care, with clinicians inquiring about constipation and recommending dietary modifications, probiotics, and physical activity. Women with specific risk profiles—particularly those with children, higher BMI, longer cycles, and lower socioeconomic status—require proactive mental health support. School-based and community menstrual health education programs should reduce stigma and promote healthcare-seeking, while workplace and university policies should provide flexible accommodations. Healthcare systems must develop care pathways enabling timely access to gynecological care, potentially through dedicated women’s health clinics or telemedicine, to prevent the 15% emergency visit rate observed in this study.

### 4.8. Limitations and Future Directions

This study has several limitations. The cross-sectional design precludes causal inference regarding directionality between dysmenorrhea and mental health. Self-reported data may introduce recall and social desirability bias. The sample size was determined by using national estimates, but because of convenience sampling, the calculated margin of error may not accurately reflect the true precision. An important limitation of this study concerns online convenience sampling. Since the questionnaire was distributed via digital platforms, the sample predominantly represents younger, urban, and highly educated population and does not reflect broader Saudi female population. The results of the study do not capture a full range of generalizability due to oversights including certain demographics such as older, rural, or less educated women. Therefore, the impact of the specific demographic groups may yield different outcomes or risk factors. This selection bias could also lead to over- or underestimation of the strength of associations. It is therefore appropriate to limit interpretation of the results to the sampled population. Due to incomplete differentiation between clinically primary and secondary dysmenorrhea might have led to the presumption in the results of primary dysmenorrhea’s prevalence and severity being much higher than reality. This likely misclassification of participants with previous gynecological surgeries or other comorbidities could be the reason of the excessive assumed burden of primary mechanisms. Additionally, the ethnic composition of the sample was not documented, and therefore the diversity of participants could not be explored. This demonstrates a limitation, as potential ethnic differences in the outcomes studied could not be explained. Furthermore, baseline parameters for the subgroups were not separately mentioned. As a result, the selection of covariates for subgroup analyses was based on variables identified in the overall sample. This fact limits the ability to fully address the potential discrepancies across subgroups. Future research should employ longitudinal and interventional designs to establish causality, incorporate objective biomarkers and imaging to improve diagnostic accuracy, use representative sampling methods, and evaluate effectiveness of multimodal interventions targeting pain, gastrointestinal symptoms, and mental health to inform evidence-based management strategies.

## 5. Conclusions

This study demonstrates that dysmenorrhea is highly prevalent among Saudi women, with pain severity—rather than mere presence—driving mental health burden. Severe pain (VAS 7–10) was associated with substantially elevated depression, anxiety, and stress scores. Constipation emerged as the strongest symptom-related predictor across both mental health and quality of life outcomes, highlighting the gut-brain axis’s role and suggesting novel therapeutic targets. Risk factors included having children, higher BMI, and longer cycles, while higher socioeconomic status and later menarche were protective. Quality of life impairment was most pronounced at moderate pain levels and associated with constipation, abdominal bloating, and prior gynecological surgery. These findings underscore the need for integrated biopsychosocial care addressing pain severity, gastrointestinal symptoms, and mental health, with targeted support for high-risk populations. Future longitudinal and interventional research is essential to guide evidence-based dysmenorrhea management in Saudi Arabia.

## Figures and Tables

**Table 1:** Sociodemographic Characteristics of Study Participants (n = 950).

Characteristics	Mean ± SD/Frequency (%)
**Age (years)**	28 ± 9.5
**BMI (kg/m^2^)**	24 ± 5.8
**Education level**	
High School Degree or Lower	340 (36%)
Bachelor’s degree	504 (53%)
Postgraduate Studies	106 (11%)
**Employment status**	
Employed	322 (34%)
Student	457 (48%)
Unemployed	171 (18%)
**Marital status**	
Non-Married/Engaged	518 (55%)
Married	386 (41%)
Divorced/widow	46 (4%)
**Number of children**	
0	574 (60%)
1	95 (10%)
2	110 (12%)
≥3	171 (18%)
**Monthly income (SR)**	
Less than 5000	557 (59%)
5000–9999	179 (19%)
10,000–14,999	107 (11%)
≥15,000	107 (11%)
**Residence**	
Rural	196 (21%)
Urban	754 (79%)

Abbreviations: SD, standard deviation; BMI, body mass index; SR, Saudi Riyal.

**Table 2:** Health Status and Lifestyle Factors (n = 950).

Characteristics	Frequency (%)
**Smoking status**	
Yes	50 (5%)
**Smoking method**	
Cigarettes	8 (1%)
Shisha/Hookah/Waterpipe/Vape	45 (5%)
**Physical activity**	
Yes	329 (35%)
**Blood-related disorder**	
Thalassemia	63 (7%)
Sickle cell anemia	105 (11%)
**Gynecological surgery history**	
Yes	157 (17%)

**Table 3:** Menstrual Cycle Characteristics (n = 950).

Characteristics	Mean ± SD/Frequency (%)
**Mean age at menarche (years)**	12 ± 2
**Mean cycle length (days)**	27 ± 7.4
**Mean bleeding duration (days)**	5.2 ± 2.2
**Menstrual blood flow**	
Heavy (>1 fully soaked pad/2 h)	97 (10%)
Moderate (>1 soaked pad/3 h)	587 (62%)
Light (<1 soaked pad/3 h)	266 (28%)
**Period regularity**	
Regular	817 (86%)
Irregular	133 (14%)
**Dysmenorrhea**	
Yes	830 (87%)
No	120 (13%)

Abbreviations: SD, standard deviation.

**Table 4:** Dysmenorrhea Characteristics and Associated Symptoms (n = 950).

Characteristics	Frequency (%)
**Pain severity (VAS)**	
Mild (1–3)	131 (14%)
Moderate (4–6)	365 (38%)
Severe (7–10)	454 (48%)
**Individual pain scores:**	
1	41 (4%)
2	37 (4%)
3	53 (6%)
4	81 (9%)
5	156 (16%)
6	128 (13%)
7	195 (21%)
8	152 (16%)
9	56 (6%)
10	51 (5%)
**Pain frequency**	
Once every 6 months	208 (22%)
Once every 3 months	271 (28%)
Every cycle	471 (50%)
**Pain management**	
No medication	420 (44%)
Over-the-counter medication	477 (50%)
Emergency department visit required	53 (6%)
**Emergency visits (ever)**	
Yes	140 (15%)
**ED visit frequency**	
Once	100 (11%)
Twice	53 (6%)
Three times or more	69 (7%)
**Pain onset timing**	
2–3 days before menstruation	366 (39%)
First day of menstruation	400 (42%)
Second–third day of menstruation	108 (11%)
No pain	76 (8%)
**Pain duration**	
One day	352 (37%)
Two to three days	464 (49%)
More than 3 days	74 (8%)
No pain	60 (6%)
**Associated symptoms**	
Mood swings	750 (79%)
Abdominal bloating	574 (60%)
Diarrhea	295 (31%)
Constipation	257 (27%)

VAS, Visual Analogue Scale; ED, Emergency Department.

**Table 5:** Mental Health and Quality-of-Life Outcomes.

Mental Health Measure (DASS-21)	Mean ± SD	Severity Categories	Frequency (%)
**Depression score**	19 ± 12	Normal	250 (26%)
		Mild	81 (9%)
		Moderate	175 (18%)
		Severe	149 (16%)
		Very Severe	295 (31%)
**Anxiety score**	16 ± 11	Normal	315 (33%)
		Mild	84 (9%)
		Moderate	181 (19%)
		Severe	178 (19%)
		Very Severe	192 (20%)
**Stress score**	22 ± 12	Normal	168 (18%)
		Mild	74 (8%)
		Moderate	172 (18%)
		Severe	130 (14%)
		Very Severe	406 (43%)
**Quality of Life score (MQLI)**	5.7 ± 2.4	—	—

SD, standard deviation; DASS-21, Depression Anxiety Stress Scale-21; MQLI, Multicultural Quality of Life Index.

**Table 6:** Univariate Associations of Dysmenorrhea with Mental Health.

Characteristic	n	Depression	Anxiety	Stress	Quality of Life
		Mean ± SD	Mean ± SD	Mean ± SD	Mean ± SD
**Dysmenorrhea status**					
No	120	15.2 ± 10.5	13.8 ± 9.8	19.1 ± 11.2	6.1 ± 2.2
Yes	830	19.6 ± 12.3	16.5 ± 11.2	22.5 ± 12.1	5.6 ± 2.4
***p*-value**		**0.001**	**0.018**	**0.006**	0.062
**Pain severity**					
Mild (VAS 1–3)	131	14.8 ± 10.8	13.2 ± 9.5	18.7 ± 10.9	6.2 ± 2.3
Moderate (VAS 4–6)	365	17.9 ± 11.5	15.1 ± 10.6	21.0 ± 11.8	5.9 ± 2.4
Severe (VAS 7–10)	454	21.8 ± 12.6	18.4 ± 11.5	24.2 ± 12.3	5.3 ± 2.4
***p*-value**		**<0.001**	**<0.001**	**<0.001**	**<0.001**
**Pain frequency**					
Occasional	479	17.2 ± 11.8	14.9 ± 10.5	20.5 ± 11.6	5.9 ± 2.4
Every cycle	471	21.1 ± 12.4	17.5 ± 11.6	23.8 ± 12.3	5.4 ± 2.4
***p*-value**		**<0.001**	**<0.001**	**<0.001**	**0.008**
**Emergency visits**					
No	810	18.5 ± 11.9	15.6 ± 10.9	21.7 ± 12.0	5.8 ± 2.4
Yes	140	22.7 ± 13.1	18.9 ± 11.8	24.5 ± 12.5	5.2 ± 2.3
***p*-value**		**<0.001**	**0.003**	**0.018**	**0.010**

SD, standard deviation; VAS, Visual Analogue Scale; *p*-values from independent *t*-tests (dysmenorrhea status, pain frequency, emergency visits) and one-way ANOVA (pain severity); Bold *p*-values indicate statistical significance (*p* < 0.05).

**Table 7:** Multivariate Analysis: Dysmenorrhea-Related Predictors of Mental Health and Quality of Life.

Predictor	Depression	Anxiety	Stress	Quality of Life
	β (95% CI)	β (95% CI)	β (95% CI)	β (95% CI)
**Pain severity** (ref: Mild, VAS 1–3)				
Moderate (VAS 4–6)	3.27 (0.38 to 6.16) *	2.64 (−0.09 to 5.37)	2.99 (0.28 to 5.70) *	−0.57 (−1.17 to 0.03)
VAS 7	6.27 (2.18 to 10.35) **	6.12 (2.50 to 9.74) **	6.64 (2.70 to 10.58) **	−0.87 (−1.75 to 0.01)
VAS 8	7.60 (3.40 to 11.80) ***	6.64 (2.92 to 10.36) ***	6.60 (2.55 to 10.66) **	−0.64 (−1.55 to 0.26)
VAS 9	10.95 (6.19 to 15.72) ***	9.95 (5.73 to 14.17) ***	10.34 (5.74 to 14.94) ***	−0.88 (−1.91 to 0.15)
VAS 10	8.19 (3.19 to 13.19) **	9.16 (4.73 to 13.59) ***	8.99 (4.17 to 13.82) ***	−0.37 (−1.45 to 0.71)
**Menstrual characteristics**				
Menarche age (per year)	−0.48 (−0.83 to −0.13) **	−0.32 (−0.63 to −0.02) *	−0.51 (−0.85 to −0.17) **	0.02 (−0.05 to 0.10)
Cycle length (per day)	0.17 (0.07 to 0.27) **	0.11 (0.02 to 0.20) *	0.20 (0.10 to 0.29) ***	0.01 (−0.01 to 0.04)
Period regularity [Yes]	0.65 (−1.38 to 2.68)	−0.29 (−2.09 to 1.50)	0.13 (−1.83 to 2.09)	−0.23 (−0.67 to 0.21)
**Associated symptoms**				
Abdominal bloating [Yes]	0.84 (−0.64 to 2.32)	1.28 (−0.03 to 2.59)	0.99 (−0.44 to 2.42)	−0.32 (−0.64 to −0.00) *
Mood swings [Yes]	−0.13 (−1.94 to 1.67)	−0.69 (−2.29 to 0.91)	1.30 (−0.44 to 3.05)	−0.18 (−0.57 to 0.21)
Diarrhea [Yes]	0.92 (−0.65 to 2.50)	0.58 (−0.82 to 1.98)	0.43 (−1.09 to 1.95)	−0.02 (−0.36 to 0.32)
Constipation [Yes]	4.94 (3.27 to 6.62) ***	4.79 (3.31 to 6.27) ***	3.96 (2.35 to 5.58) ***	−0.45 (−0.81 to −0.09) *
**Healthcare utilization**				
Emergency visits [Yes]	0.91 (−1.21 to 3.02)	1.39 (−0.48 to 3.26)	1.27 (−0.77 to 3.31)	−0.32 (−0.78 to 0.13)
Past gynecological surgery [Yes]	0.67 (−1.24 to 2.57)	1.97 (0.28 to 3.65) *	1.46 (−0.37 to 3.30)	−0.52 (−0.93 to −0.11) *

Models adjusted for age, marital status, income, education, employment, number of children, residence, smoking, and BMI. β, unstandardized regression coefficient; CI, confidence interval; VAS, Visual Analogue Scale. * *p* < 0.05; ** *p* < 0.01; *** *p* < 0.001.

## Data Availability

The data supporting the findings of this study are available from the corresponding author upon reasonable request, due to privacy and confidentiality restrictions.

## Abbreviations

The following abbreviations are used in this manuscript:

BMI	Body Mass Index
CI	Confidence Interval
SAR	Saudi Riyal
QoL	Quality of Life
VAS	Visual Analogue Scale
